# Engineering of Src Homology 2 Domain Leading to Sulfotyrosine Recognition With a High Affinity by Integrating a Distinctive Selection Theme and Next-Generation Sequencing

**DOI:** 10.3389/fmicb.2022.901558

**Published:** 2022-06-03

**Authors:** Dongping Zhao, Chan Li, Haoqiang Jiang, Yuqing Yin, Changjing Zhou, Haiming Huang, Yunkun Qi, Lei Li

**Affiliations:** ^1^School of Basic Medicine, Qingdao University, Qingdao, China; ^2^Department of Drug Discovery, Noventi Biopharmaceuticals Co., Ltd., Shanghai, China; ^3^Sino Genomics Technology Co., Ltd., Qingdao, China; ^4^School of Pharmacy, Qingdao University, Qingdao, China

**Keywords:** phage display, SH2 domain, next-generation sequencing, tyrosine sulfation, directed evolution

## Abstract

Tyrosine sulfation plays a vital role in various biochemical reactions. Although sulfated tyrosine (sTyr) has a similar structure to phosphotyrosine (pTyr), the number of available sTyr sites is significantly less than that of pTyr sites, mainly because of the lack of effective sTyr probes. A few sTyr binders were identified on the basis of structural similarity by engineering the pTyr-binding pocket of an Src Homology 2 (SH2) domain through phage selections against sTyr peptides. Nevertheless, they still interact with pTyr peptides with comparable affinity. This study aims to identify sTyr superbinders using the SH2 domain as a template. We created a distinctive phage selection scheme that separately covered selections against sTyr and pTyr peptides, followed by next-generation sequencing (NGS). After selections, phage pools showed strong enzyme-linked immunosorbent assay (ELISA) signal intensities for both modified peptides, indicating that the variants evolved with a high affinity for these peptides, which causes difficulty in identifying sTyr-specific binders. In contrast, NGS data from selected pools showed significant differences, suggesting the enrichment of sTyr-specific variants during selections. Accordingly, we obtained the sTyr features based on NGS data analysis and prioritized a few potential sTyr binders. The variant SH2-4 showed a stronger affinity for sTyr than pTyr and was superior to previous sTyr binders as measured by the Biolayer Interferometry assay. In summary, we described the strategy of integrating NGS data mining with a novel selection scheme to identify sTyr superbinders.

## Introduction

Protein tyrosine O-sulfation is a common type of post-translational modifications (PTMs) that occur in transmembrane and secreted proteins. Tyrosine sulfation, catalyzed by two tyrosyl protein sulfotransferase enzymes TPST-1 and TPST-2, plays a vital role in several cell signal pathways and biochemical reactions (Moore, 2003, 2009; Leung et al., 2016; Naider and Anglister, 2018). In AIDS, the interaction between HIV and CCR5 is affected by tyrosine sulfation of CCR5 as the mutation of sulfated tyrosine (sTyr) to phenylalanine significantly reduces HIV infection rates (Choe et al., 1996).

**FIGURE 1:**
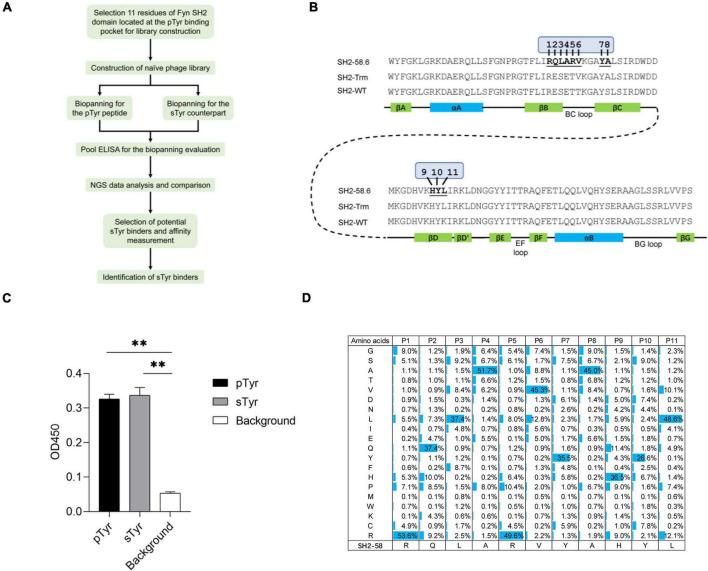
The construction and quality control of a Fyn Src Homology 2 (SH2) domain variant library. **(A)** A workflow diagram of the selection of the sulfated tyrosine (sTyr) binders. **(B)** The amino acid sequence and secondary structure of the Fyn SH2 domain (SH2-WT), the SH2 superbinder (SH2-trm) for phosphotyrosine (pTyr) (Kaneko et al., 2012), and the variant SH2-58.6 as the library template in this study (Ju et al., 2016). The 11 evolvable residues were numbered on SH2-58.6. **(C)** Cross-reactivity of SH2-58.6 binding to pTyr peptide and sTyr counterpart measured by phage enzyme-linked immunosorbent assay (ELISA). Streptavidin precoated in the microplate was taken as the background. Three replicates were performed, and the *p*-value was performed by Student’s *t*-test (^**^p < 0.01). **(D)** The amino acid distribution at evolvable positions was calculated based on the next-generation sequencing (NGS) data. The corresponding residues of the template SH2-58.6 were shown at the bottom.

Up to 1% of tyrosine residues in animals are predicted to be sulfated (Huttner, 1988). Due to the lack of biomolecular probes to effectively enrich sulfated tyrosine, several sTyr-containing proteins or sTyr sites have been reported. Available anti-sTyr antibodies are specific for sTyr over phosphorylated tyrosine (pTyr), but their binding affinities are moderate (IC50 = 1 μM or 1.25 mM) (Hoffhines et al., 2006; Kehoe et al., 2006). In contrast, although sTyr and pTyr have a similar structure (Supplementary Figure 1), anti-pTyr antibodies have an excellent affinity for pTyr, and tens of thousands of pTyr sites have been documented (Hornbeck et al., 2019). Recently, the Src Homology 2 (SH2) domain (about 100 amino acids) has been engineered as an SH2 superbinder (i.e., SH2-trm; Kaneko et al., 2012; Figure 1B) by phage display to recognize phosphotyrosine (pTyr) through the evolution of its pTyr-binding pocket and has been shown to be superior to anti-pTyr antibodies (Bian et al., 2016). Using structural similarities between phosphorylation and sulfation (Supplementary Figure 1), the pTyr-binding pocket of SH2-trm was engineered to identify sTyr (Ju et al., 2016; Lawrie et al., 2021). A couple of SH2 variants (e.g., SH2-58.6, Figure 1B) were reported to bind to sTyr, but they also recognize pTyr with a moderate affinity (Ju et al., 2016; Lawrie et al., 2021; Li et al., 2021). All of the variants described above are derived from the human Fyn or Src SH2 domain, both belonging to the Src family and with high homology (e.g., 66% sequence identity) (Liu et al., 2006).

In this study, we engineered the sTyr binder SH2-58.6 to identify SH2 variants with improved affinity and specificity for sTyr. Our strategy is illustrated in Figure 1A. There are three differences between this study and previous studies. First, previous sTyr binders were developed by randomizing amino acid residues at four or five positions in the pTyr-binding pocket (Ju et al., 2016; Lawrie et al., 2021). In contrast, we increased the number of evolvable positions to 11 because more variable residues might result in variants of higher affinity and specificity. Second, the reported sTyr binders evolved through positive selections against sTyr peptides or integrated with negative selections against the pTyr counterpart. As sTyr binders interact with both sTyr and pTyr peptides, we doubt the effectiveness of these selection strategies. Accordingly, we separately developed a distinctive phage selection theme, which included positive selections against both sTyr and pTyr peptides. Variants that were enriched during selections on sTyr but depleted during selections against pTyr were considered to have a high affinity and specificity for sTyr. Third, in previous studies, sTyr binders were identified using random selections of clones from the phage pool, so that variants with high specificity might be omitted. We performed next-generation sequencing (NGS) of the rounds of selections and performed NGS data analysis to prioritize variants. Finally, we identified the SH2 variant SH2-4, which has improved affinity and specificity for sTyr and is superior to the documented sTyr binders. In summary, we described the strategy for integrating the novel selection scheme and NGS and model construction to identify sTyr superbinders. We expect that they will be used to enrich sTyr peptides and boost the study of tyrosine sulfation on a proteomic scale.

## Results

### Construction of the SH2 Domain Library and Its Quality Control by Next-Generation Sequencing

Two SH2 variants (SH2-58.6 and SH2-60.1) were documented as specific for sTyr over pTyr (Ju et al., 2016). We selected the variant SH2-58.6 as a template for constructing the SH2 domain library. We generated Fyn SH2-58.6 (Figure 1B) in the pFN-OM6 phagemid and found that this variant could bind to pTyr and sTyr peptides (Figure 1C). Next, we randomized 11 positions on the pTyr-binding pocket, including those selected for recognizing sTyr binders (Ju et al., 2016; Lawrie et al., 2021), to identify SH2 variants with improved affinity and specificity for sTyr. By choosing more variable positions than before, we attempted to understand how pocket residues evolved to allow tighter binding to a sTyr ligand. To this end, residues at these positions were systematically altered using the soft randomization mutagenesis technique (Li et al., 2021), which allows a 50% mutation rate at each position. The library capacity estimated by monoclonal titration is about 1.0 × 10^9^. This library was defined as the naïve library for the following biopanning.

We amplified the DNA sequences of SH2 variants and performed NGS to characterize the library. As a result, 1.62 million high-quality DNA sequences coding Fyn SH2 variants were retrieved from the sequencing data. Of these sequences identified, 1.54 million (95%) have the designed mutations in 11 evolvable positions, while the rest of them have unexpected mutations, likely due to mutations introduced by PCR amplification or sequencing errors. Further analysis of the amino acid distribution of Fyn SH2 variants showed that the actual mutation of the variant library was in line with the theoretical design with minor exceptions, as shown in Figure 1D.

### Biopanning

This study used two modified peptides to identify sY superbinders for biopanning: sY peptide and pY counterpart. sY peptide was used to discover sY superbinders from SH2 domain-based phage libraries (Ju et al., 2016; Lawrie et al., 2021). Similarly, pY peptide was employed to explore pY superbinders from SH2 domain-based phage libraries (Li et al., 2021). Therefore, we selected these two modified peptides and constructed an SH2 domain-based phage library for this study.

In previous studies, SH2 variants as sTyr binders evolved by positive selections against sTyr peptide alone or in combination with negative selections against pTyr counterpart (Ju et al., 2016; Lawrie et al., 2021). These selection schemes are the common strategies based on the hypothesis that specific variants of sTyr rather than pTyr are enriched during selections. Nevertheless, all identified sTyr binders can bind pTyr with moderate affinities (Ju et al., 2016; Lawrie et al., 2021). This indicates that SH2 variants that bind to both modifications are challenging to filter out using such selection schemes. To solve this problem, we developed a novel selection theme to find SH2 variants with a superior affinity and specificity for sTyr. This novel selection theme included selections against both pTyr and sTyr peptides separately, NGS sequencing of the selection results, and the comparison between their NGS data to prioritize potential sTyr binders with a high affinity. We hypothesized that such sTyr binders were depleted in the process of selections against the pTyr target but enriched in the selections against the sTyr target.

**FIGURE 2:**
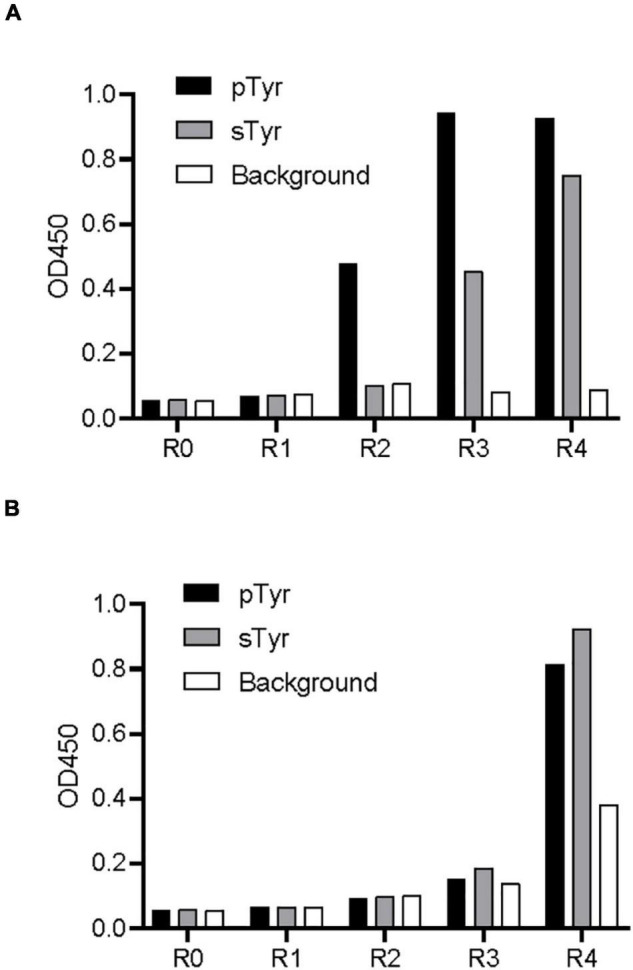
Biopanning of the SH2 variant library against pTyr and sTyr peptides. **(A)** Phage ELISA of the selected phages from each biopanning round against pTyr and the naïve library binding to pTyr peptide and sTyr counterpart. R0 refers to the naïve library. R1–R4 stands for phage panning cycle Rounds 1–4. **(B)** Phage ELISA of the selected phages from each biopanning round against sTyr and the naïve library binding to pTyr peptide and sTyr counterpart. Streptavidin precoated in the microplate was taken as the background.

The library was subjected to phage biopanning against a biotinylated pTyr peptide derived from the protein MidT, a cognate ligand of wild-type Fyn SH2 (Dunant et al., 1997). The phage library was preincubated with the biotin-labeled pTyr peptide, which was immobilized in a microplate precoated with streptavidin. Bound phages were eluted, amplified, and applied as input to the next round of panning. After four rounds of panning, the amplified phage pools from these rounds were applied to an enzyme-linked immunosorbent assay (ELISA) to test their binding to pTyr peptide and sTyr counterpart. Figure 2A shows that the phages are explicitly bound to pTyr peptide after the second round and interact with pTyr and sTyr peptides after the third and fourth rounds. This indicates that SH2 variants that interact with both modified peptides were enriched in the round of panning.

Similarly, the library was subject to phage biopanning against the biotinylated sTyr peptide. Figure 2B shows that phage pools did not bind to either peptide until the fourth round, at which interactions of the phages with both modified peptides had similar signal intensities. Unexpectedly, the apparent background signal intensity in the fourth round indicates that non-specific phages that bind to the background was also enriched (Figure 2B). The enrichment of non-specific phages and those bound to both modified targets suggest the difficulty of finding sTyr-specific binders through random selections from pools.

### Computational Analysis of Next-Generation Sequencing Data to Prioritize Potential Sulfated Tyrosine Binders

**FIGURE 3:**
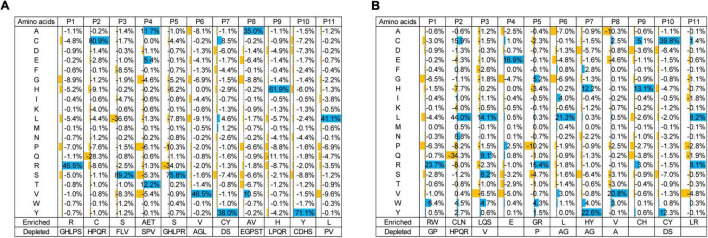
Proportion changes of residue types at evolvable positions from the naïve library to the library at the fourth round of selection against pTyr **(A)** and sTyr **(B)**. Residue types with significant proportion changes (5% as cutoff) were considered enriched or depleted and were listed at the bottom.

Enzyme-linked immunosorbent assay showed similar intensities of pTyr and sTyr binding to phage pools in the fourth selection against the pTyr target and the sTyr counterpart (Figure 2). Such observations raised the question, “what are the differences between the phage pools biopanned using pTyr and sTyr targets?” We figured it out by performing NGS of phage-displayed SH2 variants panned from all rounds. First, we investigated the residue types that were enriched or depleted by panning against the pTyr target. To this end, we calculated proportion changes in the residue type at each evolvable position from the naïve library to the library after the fourth round of selection (Figure 3A). The residue type was defined as enriched if its proportion change was greater than 5% or depleted if it was less than −5%. Figure 3A shows the enrichment or depletion of various residue types at different positions. The results were similar if the naïve library was compared with the library after the third round (Supplementary Figure 2). Next, after the fourth round, we performed a similar analysis for panning against the sTyr target by comparing the naïve library with the selected library. Similarly, many residue types were enriched or depleted at distinct positions. A comparison of Figures 3A,B illustrated that many residue types were enriched for selections against both modified targets, whereas a few showed the opposite trend. For instance, the amino acids R@P1, C@P2, S@P3, E@P4, Y@P7, V@P8, H@P9, Y@P10, and L@P11 were enriched in both selections (Figure 3). In contrast, the amino acid S@P5 was enriched by panning against pTyr but depleted by panning using sTyr, while the amino acid L@P6 was the opposite (Figure 3). These observations indicate that while both phage libraries panned against sTyr and pTyr have similar enriched residues at many positions, each library has its characteristics. Therefore, SH2 variants specific to sTyr are likely to exist in the library panned against sTyr.

We hypothesized that sTyr-specific binders had three features: (1) their percentages in the pool tend to increase during selections against sTyr but decrease for selections against pTyr; (2) they are liable to include a large number of residues enriched in selections against sTyr; and (3) the number of such enriched residues is more significant than that of enriched residues in selections against pTyr. We took advantage of the NGS data to identify variants with these features. Practically, we only considered variants as potential sTyr-specific binders whose proportion was larger than 0.1% at the last few rounds of selection against sTyr. Additionally, we defined the term “enrichment index (EI)” to quantify the enrichment status of a given variant. For each position, an enriched residue type (proportion change > 5%) corresponds to the value of 1, a depleted residue type (proportion change < −5%) is related to −1, and the other residue types are 0 (Figures 3A,B). The EI value is the sum of the values at the evolvable positions. For instance, the variant with “RCSEGLYVHYL” had the sTyr EI value as 11 but 8 for pTyr (Figures 3A,B). A potential sTyr-specific binder is likely to have a considerable sTyr EI value, but a small pTyr EI value. In other words, such a binder has a significant EI difference between the sTyr and pTyr EI values. Accordingly, we used the sTyr EI value and EI difference and the tendency of the proportion in selections to prioritize SH2 variants.

**FIGURE 4:**
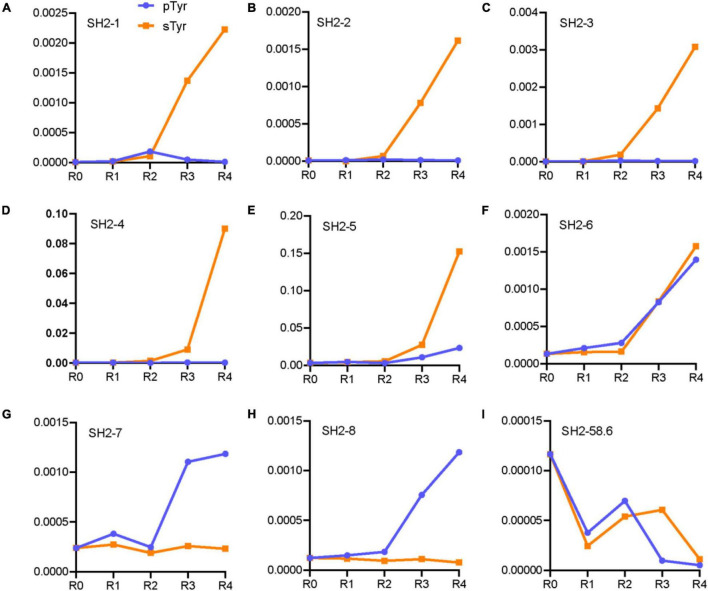
Line chart of proportions of the variants in the rounds of selections against pTyr and sTyr peptides. These variants are SH2-1 **(A)**, SH2-2 **(B)**, SH2-3 **(C)**, SH2-4 **(D)**, SH2-5 **(E)**, SH2-6 **(F)**, SH2-7 **(G)**, SH2-8 **(H)** and SH2-58.6 **(I)**.

We predicted and selected 12 variants to examine their affinity for pTyr and sTyr peptides. These variants were classified into three categories: the first four (SH1-1–SH2-4) were potential sTyr-specific binders, the second four (SH2-5–SH2-8) were sTyr-specific non-binders, and the remaining four (SH2-58.6 to SH2-WT) were derived from the literature (Ju et al., 2016; Lawrie et al., 2021) for comparison (Table 1). SH1-1–SH2-4 variants had large sTyr EI values (9 or 10) and EI differences (ranging from 5 to 9), and their proportions constantly increased during selections against sTyr but decreased or had a slight change in selections against pTyr (Figures 4A–D). Therefore, these variants were predicted as potential sTyr binders. The variants SH2-5–SH2-8 have minor EI differences (−1 to 2) (Table 1). The percentages of SH2-5 and SH2-6 increased in selections against sTyr and pTyr. The percentages of SH2-7 and SH2-8 increased during selections against pTyr but decreased or slightly changed against sTyr (Figures 4E–H). Therefore, SH2-5–SH2-8 were not thought of as potential sTyr-specific binders. Moreover, the template SH2-58.6 had the EI difference of 0, and its proportions generally decreased during selections against sTyr and pTyr, indicating that it was overcome during selections. Furthermore, we failed to find sTyr binders (i.e., SH2-1.8 and SH2-3.1) and the wild-type SH2 domain documented in the NGS data. They all had slight EI differences (from −2 to 1), so they were not considered sTyr-specific binders.

**TABLE 1:** Residues at the evolvable positions and EI values and binding affinities of Src Homology 2 (SH2) variants to phosphotyrosine (pTyr) and sulfated tyrosine (sTyr) peptides.

SH2^#^	P1	P2	P3	P4	P5	P6	P7	P8	P9	P10	P11	Detected in NGS?	EI		EI difference^$^	Kd(nM)	
													(sTyr)	(pTyr)		(sTyr)	(pTyr)
**Potential sTyr binders**																	
SH2-1	R	L	L	A	R	L	Y	A	H	C	L	Yes	10	2	8	No expressed	
SH2-2	R	L	L	A	R	V	Y	A	H	C	L	Yes	9	4	5	No expressed	
SH2-3	R	L	L	A	R	L	Y	A	Q	C	L	Yes	9	0	9	No expressed	
SH2-4	R	N	Q	P	G	V	Y	V	H	Y	R	Yes	9	4	5	97.9	131
**Other variants**																	
SH2-5	R	C	S	E	R	V	H	V	H	Y	L	Yes	10	8	2	117	55.3
SH2-6	R	C	S	A	R	V	H	V	H	Y	L	Yes	9	8	1	126	65.4
SH2-7	R	Q	S	A	R	V	F	V	H	Y	L	Yes	6	6	0	145	55.5
SH2-8	R	C	S	E	R	R	Y	V	H	Y	L	Yes	10	8	2	147	61.6
**Reported sTyr binders or SH2 wildtype**																	
SH2-58.6	R	Q	L	A	R	V	Y	A	H	Y	L	Yes	5	5	0	188	141
SH2-1.8	R	E	A	E	R	V	Y	A	H	Y	L	No	6	7	−1	266	215
SH2-3.1	R	E	H	P	F	V	Y	A	H	Y	L	No	4	6	−2	282	163
SH2-WT	R	E	S	E	T	T	Y	S	H	Y	L	No	6	5	1	ND	267

*^#^SH2 variants were classified into three categories: (1) potential sTyr binders, (2) other variants, and (3) reported sTyr binders plus wild-type SH2 domain.*

*^$^The EI difference means the difference between the sTyr EI value and the pTyr EI value. EI means enrichment index.*

*ND, No detected.*

### SH2-4 Had a Stronger Affinity to Sulfated Tyrosine Than Phosphotyrosine and Was Superior to Others

**FIGURE 5:**
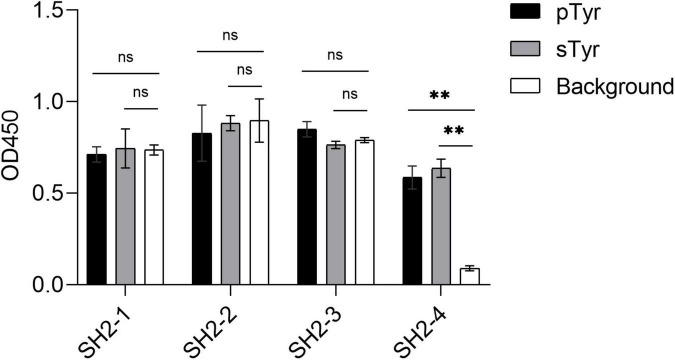
Phage ELISA of the variants SH2-1–SH2-4 binding to pTyr and sTyr peptides and the background. Streptavidin precoated in the microplate was taken as the background. Three replicates were performed, and the *p*-value was performed with Student’s *t*-test (ns, no significant; ^**^p < 0.01).

Using a Biolayer Interferometry assay, we expressed 12 SH2 variants and measured their affinities for the sTyr and pTyr peptides. Interestingly, we successfully expressed nine of them *via* the expression vector pHH0239, except SH2-1–SH2-3. To determine why the three variants failed to express, we attempted to express SH2-1–SH2-4 using the phagemid pFN-OM6 and measure their binding to the modified peptides through phage ELISA. The variants SH2-1–SH2-3 were expressed successfully but were bound to the background, whereas SH2-4 did not interact with the background (Figure 5). As they had the common C@P10, we suspected that C@P10 was the reason for solid background ELISA signals in selections against sTyr and explored its association with background signals accordingly (Figures 2, 4). The proportion of C@10-containing variants increased from 7.8% in the naïve library to 47.6% in the fourth round of selection against sTyr and was significantly correlated with background signal intensities (Pearson’s *r* = 0.88). In contrast, such a proportion decreased to 0.5% in selections on pTyr, and background signal intensities were constantly low. These observations suggest that C@P10 may bind to the background and result in non-specific binding.

As the last potential sTyr binders, SH2-4 showed a strong affinity for sTyr peptide (*K*_*d*_ = 97 nM) than pTyr peptide (*K*_*d*_ = 131 nM) (Table 1 and Supplementary Figure 3A). Four variants in the second category had weaker affinities for sTyr (*K*_*d*_ = 117–147 nM) than pTyr (*K*_*d*_ = 55–60 nM) (Supplementary Figures 3B–E). In the third category, the three documented sTyr binders had a weaker affinity for sTyr (*K*_*d*_ = 190–280 nM) than pTyr (*K*_*d*_ = 140–215 nM) (Supplementary Figures 3F–H); SH2-WT bound to pTyr peptide with the affinity *K*_*d*_ as 267 nM but failed to interact with sTyr peptide (Supplementary Figure 3J). Therefore, our data analysis method to prioritize sTyr binders is effective. In summary, SH2-4 has a strong affinity for sTyr compared to others and high specificity for sTyr. As previous sTyr binders have been shown to be useful for detecting and enriching sulfoproteins, SH2-4 can potentially be applied to the study of protein tyrosine O-sulfation with proper experimental designs.

Nature commonly utilizes multiple binding sites to interact with targets, including tandem reader domains (Moore et al., 2013). In a previous work where domains were used as detection reagents, the fusion of multiple copies of the same domain was employed to increase the affinity (Albanese et al., 2020). Accordingly, we explored whether linking multiple copies of SH2-4 would provide additional improvements beyond a single copy. Accordingly, two SH2-4 were connected using the Gly4Ser linker. As expected, the tandem SH2-4 had a higher affinity for both modified peptides (*K*_*d*_: sTyr = 74 nM; pTyr = 90 nM) than for the single SH2-4 (Figure 3I). It demonstrates that multiplexing modules can enhance the binding to the target.

## Discussion

In this study, the variant SH2-4 was found to be a sTyr superbinder because it had a high affinity and specificity to sulfotyrosine compared to the documented sTyr binders. Unlike previous studies, this identification was based on a novel strategy that integrates different library constructs, distinctive selection schemes, NGS, and model construction to identify sTyr superbinders. Previous sTyr binders (Ju et al., 2016; Lawrie et al., 2021) were based on the sequence of the SH2 superbinder SH2-trm (Figure 1B; Kaneko et al., 2012) that had a super affinity for pTyr due to the three critical residues (V@P6, A@P8, and L@P11) but had a weak affinity for sTyr (Ju et al., 2016; Lawrie et al., 2021). These critical residues were fixed in sTyr binders, indicating their reliance on the binding mechanism of SH2-trm. They were developed by randomizing four or five positions (P1–P5) to change the specificity of SH2-trm to sTyr (Ju et al., 2016; Lawrie et al., 2021). In contrast, the two critical residues of SH2-trm were mutated in SH2-4 (A8V and L11R), suggesting that SH2-4 has a different binding mechanism from previous sTyr binders. In addition, the residues at the randomized positions of previous sTyr binders were mutated in SH2-4 (Q2N, L3Q, A4P, and R5G), and none of them were identical to previous sTyr binders, suggesting that SH2-4 has a different specificity mechanism. In summary, SH2-4 and previous sTyr binders have different binding and specificity mechanisms.

We previously built a phage library by randomizing several positions in the pTyr-binding pocket of the SH2 domain and identified SH2 superbinders after a few rounds of selections against pTyr peptides (Kaneko et al., 2012; Li et al., 2021). As some of them were found to interact with sTyr peptides (Ju et al., 2016; Lawrie et al., 2021), we thought that the variants bound to pTyr and sTyr peptides were enriched during selections against pTyr. This thought was proven in this study (Figure 2A). This observation is comprehensible as sTyr and pTyr have structural similarities (Supplementary Figure 1). Therefore, it is expected that variants bound to pTyr and sTyr peptides were also enriched during selections against sTyr in this study (Figure 2B). Despite this, pTyr-specific variants are likely to be enriched in selections against pTyr, and sTyr-specific variants tend to be enriched in selections on sTyr. To find sTyr-specific variants effectively, we did not use the random phage selection technique but performed NGS for all rounds of selections, followed by a systematic comparison of the variants enriched in selections against pTyr with those enriched in the selection against sTyr. NGS data analyses showed that the variants enriched in the selected pools had different characteristics (Figures 3A,B), indicating the existence of variants with high specificity for sTyr. Accordingly, we prioritized the variants based on the characteristics and found that SH2-4 had a superior affinity and specificity for sTyr. Nevertheless, SH2-4 still binds pTyr with a moderate affinity. In the future, we will optimize the prediction model or take SH2-4 as a template for rebuilding a phage library to identify variants with a higher affinity and specificity for sTyr.

## Materials and Methods

### Construction of the Fyn SH2 Domain Phage Display Library

Fyn SH2-58.6 was subcloned into pFN-OM6 phagemid, which was used to construct the PIII fusion protein. Fyn SH2-58.6 in pFN-OM6 was used as a template for library construction. Primer1 (AGAGGTACCTTTCTTATC**TAACCATGGTAA**CTTTCTATC CGTGATTGG) and Primer2 (AAAGGAGACCATGTCAAA **TAACCATGGTAA**ATTCGCAAACTTGACAAT) were used to construct the template “TAA CCATGG TAA” (TAA: stop codon; CCATGG: Nco1 site) *via* the Kunkel reaction (Kunkel et al., 1991). The ssDNA template was prepared for Kunkel reaction. The random mutation regions of Primer3 (AGAGGTACCTTTCTTATC**N_3_N_4_N_3_N_3_N_1_N_1_N_2_N_2_N_1_N_4_N_3_N_1_ N_3_N_4_N_3_N_4_N_2_N_1_**AAAGGTGCG**N_2_N_1_N_2_N_4_N_3_N_4_**CTTTCTATC CGTGATTGG) and Primer4 (AAAGGAGACCATGTCAAA**N_3_ N_1_N_2_N_2_N_1_N_2_N_3_N_2_N_1_**ATTCGCAAACTTGACAAT) replaced the ssDNA template using the Kunkel method. In Primer3 and Primer4, N1, N2, N3, and N4 represent the mixture of A, T, C, and G with different percentages, respectively (Supplementary Table 1). Kunkel products (heteroduplex double strand) were purified and prepared for *Escherichia coli* SS320 (Genentech) electroporation. After being cultured overnight, dsDNA was extracted from electroporated *E. coli* SS320. After that, dsDNA was digested by Nco1 (New England Biolabs) and transferred to *E. coli* SS320 (pre-infected by M13KO7) by electroporation. The electroporated *E. coli* SS320 was cultured in 2YT (10 g yeast extract, 16 g tryptone, 5 g NaCl, water was added to make up the volume to 1.0 L, adjusted pH to 7.0 with NaOH, autoclaved) at 32°C overnight. The phage display library was precipitated with PEG/NaCl (20% PEG 8000, 2.5 M NaCl) and resuspended with polybutylene terephthalate [PBT; 0.05% Tween, 0.5% bovine serum albumin (BSA) in phosphate-buffered saline (PBS)] buffer.

### Next-Generation Sequencing and Data Analysis

DNA samples from nine Fyn SH2 libraries were amplified by PCR using the forward primer (5′- GAGGTACCTTTCTTATC-3) and the reverse primer (5′-TTGTCAAGTTTGCGAAT-3′) with different labels. About > 50 ng of the purified PCR fragment was used for library preparation. These PCR products were treated with End Prep Enzyme Mix for end repair. After purification with magnetic beads, the amplified products were amplified by P5 and P7 primers with sequences that can anneal with flow cells to perform bridge PCR and index, allowing multiplexing. The library was then obtained by magnetic bead purification. The library was sequenced with a pair-end of 150 bp in the Illumina NovaSeq 6000 sequencing system. The method of data analysis was performed as described (Li et al., 2021).

This study’s raw sequencing data have been deposited in the NCBI Sequence Read Archive database (Accession No. PRJNA790495).

### The Fyn SH2 Domain Library Panning

Biopanning for negative selection against streptavidin (Solarbio) was performed by incubating a phage library (10^13^ CFU/ml) in a well of a streptavidin-coated 96-well plate (Thermo Fisher Scientific F96 Maxisorp NUNC-immuno plate, Lot No. 168854) for 1 h. Next, the phage library was separately transformed into the streptavidin-coated wells, with the immobilized 16 pmoL/well biotin-sulfopeptide (biotin-ahx-ahx-EPQsYEEIPIYL) and the corresponding wells with the immobilized biotin-phosphopeptide (biotin-ahx-ahx-EPQpYEEIPIYL). After 1 h of incubation, positive wells were washed 10 times using the PT buffer (PBS with 0.05% Tween). Bound phages were eluted by 100 mM HCl 100 μl/well and neutralized by adding 1/8 volume of Tris-HCl (1 M, pH = 11). The amplification of the phage library and its selection stringency were performed as described (Li et al., 2021).

### Phage Enzyme-Linked Immunosorbent Assay

About 6 pmoL/well streptavidin in 50 μl PBS was coated in a 96-well NUNC microplate at 4°C overnight. The next day, the solution in the wells was discarded. Around 2% milk was added to wells for blocking. After incubation for 1 h, the wells were washed three times with the PT buffer. Peptides of 8 pmoL/well (biotin-ahx-ahx-EPQ-sTyr-EEIPIYL) in PBS were added for immobilization at room temperature for 1 h. Wells containing no peptides were used as the negative control. After washing five times with the PT buffer, phages in 50 μl PBS were incubated in wells for 1 h at room temperature. Anti-M13/HRP monoclonal antibody conjugate (50 μl, 1:8,000 dilution, Sino Biological) in PBS buffer (1:8,000 dilution, 50μl) was added to the wells and incubated for 30 min. According to the manufacturer’s instructions, after washing with the PT buffer eight times, the tetramethylbenzidine (TMB) buffer (Biopanda, 50 μl/well) was added for development. Within 5 min, 50 μl/well of 1.0 M H_3_PO_4_ was added to stop the reaction. After that, OD450 values were measured.

### Protein Expression and Purification

cDNA sequences of SH2 domain variants in pFN-OM6 were subcloned into *Sfi*I and *Not*I sites of pHH0239 to express 6xHis-tag proteins at the N-terminus. The constructed pHH0239 was transformed into *E. coli* SR320. Single colonies were added to a 2YT medium containing 50 μg/ml carbenicillin and cultured at 37°C to OD600 = 0.6, followed by adding 0.5 mM IPTG (Solarbio) and induction overnight at 18°C. After that, *E. coli* SR320 was harvested by centrifugation (16,000 g, 10 min, 4°C) and lysed by sonication. Debris was removed by centrifugation (16,000 g, 30 min, 4°C). According to the manufacturer’s manual, proteins were purified by Ni-NTA agarose (Qiagen). Methods for changing elution buffer and measuring protein concentration were described elsewhere (Li et al., 2021).

### Biolayer Interferometry Assay

The Octet RED96 System (ForteBio) was used for the Biolayer interferometry assay. A streptavidin biosensor (ForteBio) was used to perform the measurement. The peptides biotin-ahx-ahx-EPQ-pTyr-EEIPIYL and biotin-ahx-ahx-EPQ-sTyr-EEIPIYL were immobilized on the biosensor. The concentration (300, 150, and 0 nM) of SH2 variants was used for the measurement. All steps were described elsewhere (Li et al., 2021).

## Data Availability Statement

The data presented in the study are deposited in the NCBI BioProject repository, accession number PRJNA790495.

## Author Contributions

LL: conceptualization, data curation, formal analysis, funding acquisition, investigation, methodology, project administration, supervision, writing—original draft, and writing—review and editing. DZ: data curation, investigation, validation, and writing—original draft. HJ: data curation and methodology. YQ: investigation, validation, and writing—original draft. HH: supervision and funding acquisition. YY and CZ: investigation. All authors contributed to the article and approved the submitted version.

## Conflict of Interest

HH and YY were the employees of Noventi Biopharmaceuticals Co., Ltd. CZ was the employee of Sino Genomics Technology Co., Ltd. LL, DZ, and CZ filed a provisional Chinese patent application related to this work. DZ was an intern of Noventi Biopharmaceuticals. The remaining authors declare that the research was conducted in the absence of any commercial or financial relationships that could be construed as a potential conflict of interest.

## Publisher’s Note

All claims expressed in this article are solely those of the authors and do not necessarily represent those of their affiliated organizations, or those of the publisher, the editors and the reviewers. Any product that may be evaluated in this article, or claim that may be made by its manufacturer, is not guaranteed or endorsed by the publisher.
