# Supplementary material for: Engineering of Src Homology 2 Domain Leading to Sulfotyrosine Recognition With a High Affinity by Integrating a Distinctive Selection Theme and Next-Generation Sequencing
Source: Front Microbiol. 2022 Jun 3;13:901558. doi: 10.3389/fmicb.2022.901558 (PMC9204161; doi:10.3389/fmicb.2022.901558)
Supplement: Supplementary file 1 [file Table_1.DOCX]

Supplementary Material

# Supplementary Figures and Tables

## Supplementary Tables

|  | A | T | C | G |
| --- | --- | --- | --- | --- |
| N1 | 70% | 10% | 10% | 10% |
| N2 | 10% | 70% | 10% | 10% |
| N3 | 10% | 10% | 70% | 10% |
| N4 | 10% | 10% | 10% | 70% |

Table S1. Nucleic acid distribution of each symbol in the primers.

## Supplementary Figures


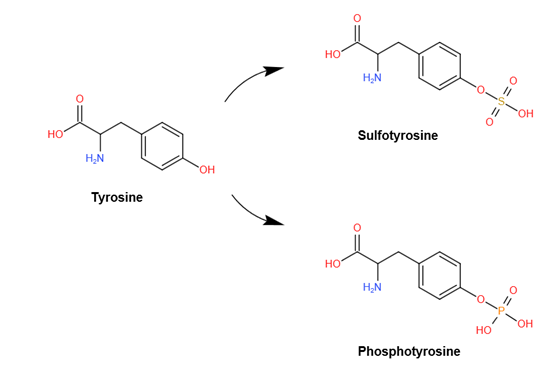


**FIGURE S1. Molecular structures of tyrosine, sulfotyrosine, and phosphotyrosine.**


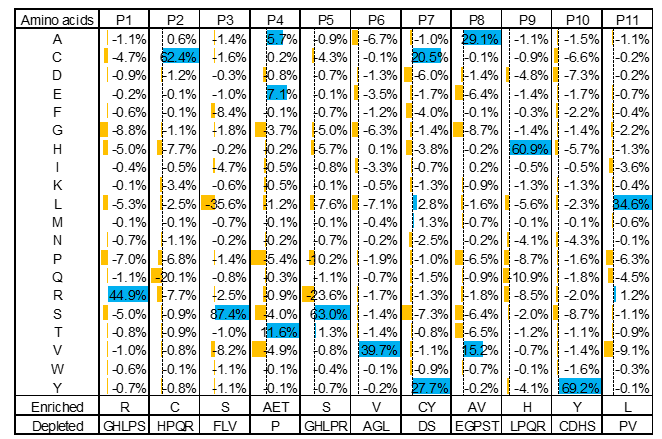


**FIGURE S2. Proportion changes of residue types at evolvable positions from the naïve library to the library at the third round of selection against pTyr.**


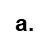

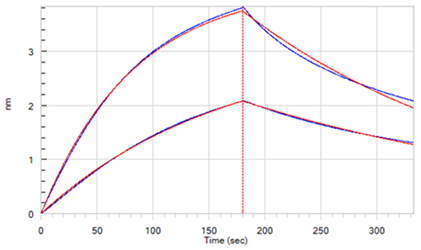

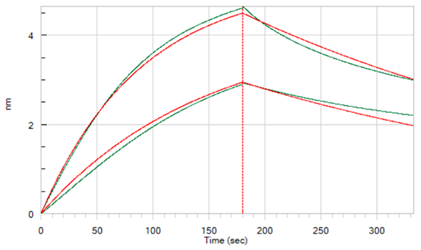


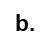

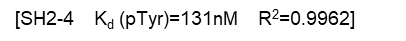

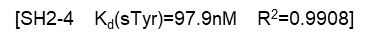


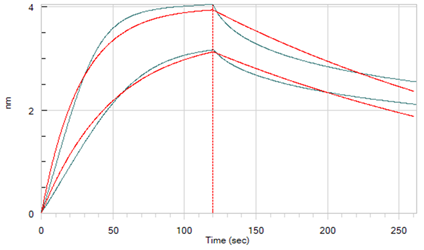

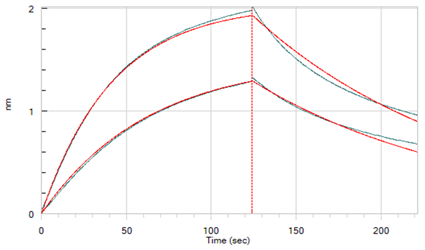


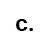

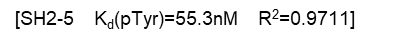

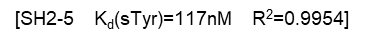


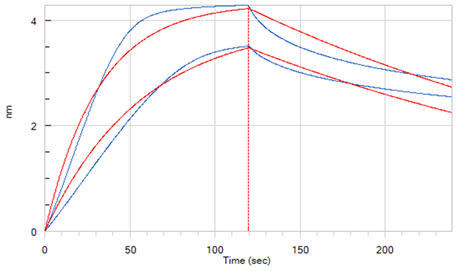

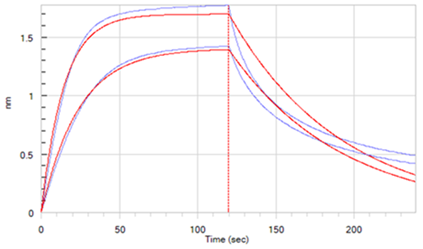


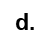

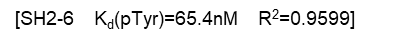

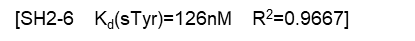


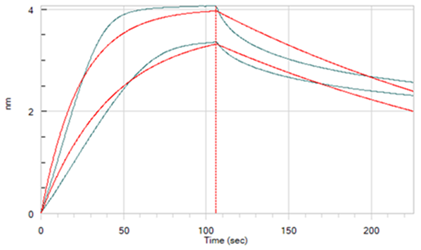

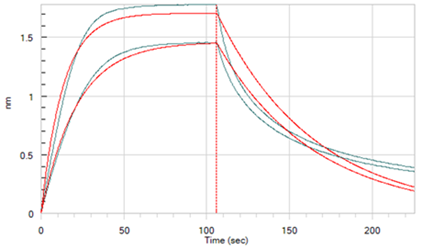


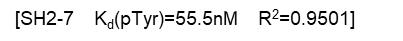

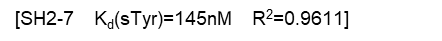


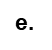

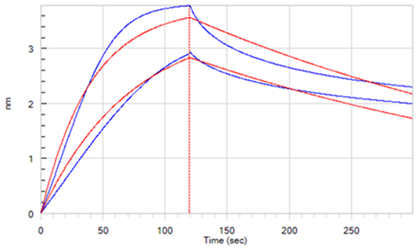

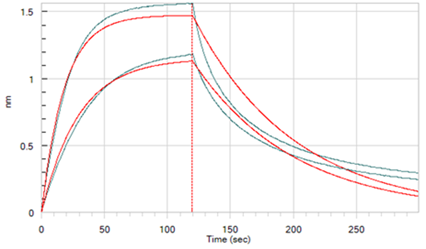


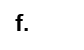

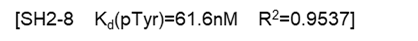

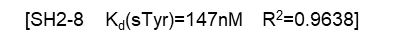


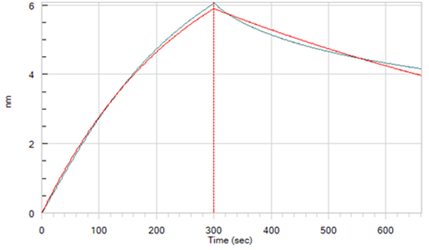

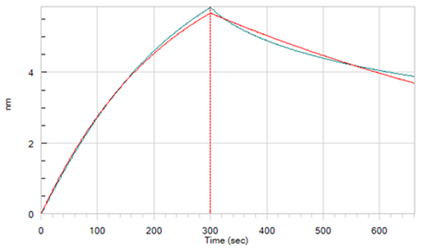


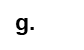

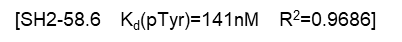

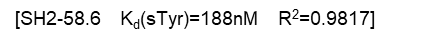


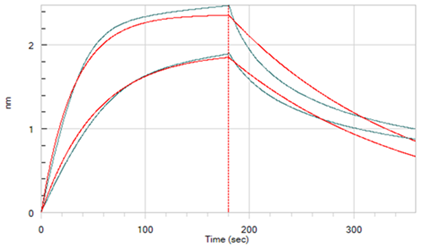

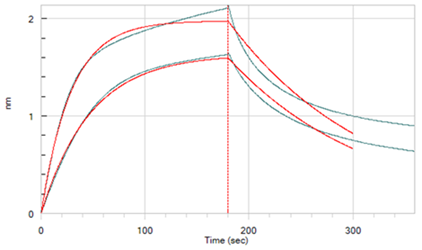


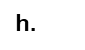

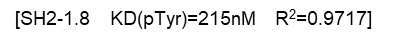

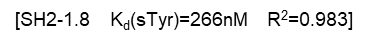


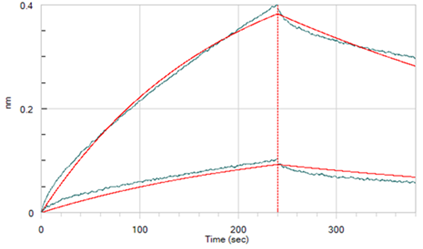

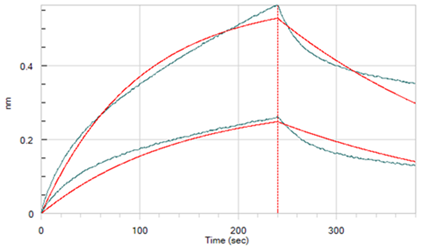


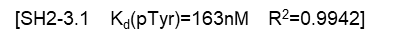

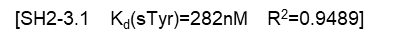


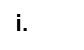

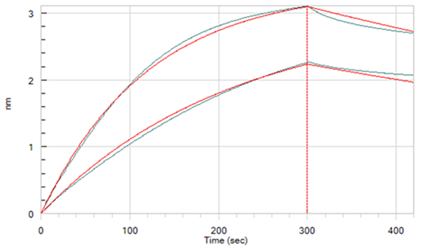

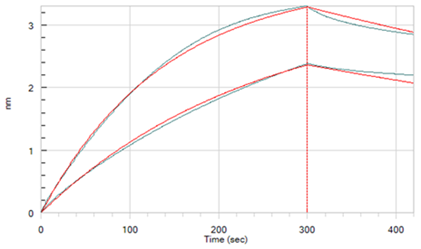


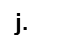

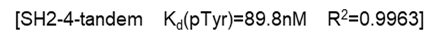

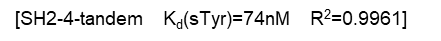


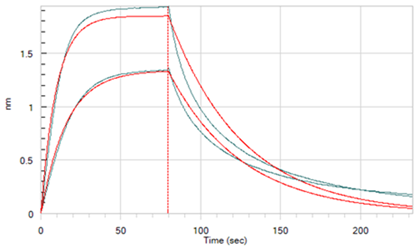


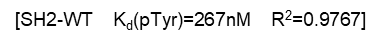


**FIGURE S3. Quantitative binding analysis of SH2 variants. Binding affinities of SH2 variants to the pTyr peptide and the sTyr counterpart, as measured by the Biolayer Interferometry assay, including (a) SH2-4, (b) SH2-5, (c) SH2-6, (d) SH2-7, (e) SH2-8, (f) SH2-58.6,(g) SH2-1.8, (h) SH2-3.1, (i) SH2-4-tandem, (j) SH2-WT.**
